# Preparation of Luminescent Metal-Organic Framework Films by Soft-Imprinting for 2,4-Dinitrotoluene Sensing

**DOI:** 10.3390/ma10090992

**Published:** 2017-08-25

**Authors:** Javier Roales, Francisco G. Moscoso, Francisco Gámez, Tânia Lopes-Costa, Ahmad Sousaraei, Santiago Casado, Jose R. Castro-Smirnov, Juan Cabanillas-Gonzalez, José Almeida, Carla Queirós, Luís Cunha-Silva, Ana M. G. Silva, José M. Pedrosa

**Affiliations:** 1 Departamento de Sistemas Físicos, Químicos y Naturales, Universidad Pablo de Olavide, Ctra. Utrera Km. 1, 41013 Sevilla, Spain; fgarciamoscoso@gmail.com (F.G.M.); fgammar@gmail.com (F.G.); tlopcos@upo.es (T.L.-C.); 2 Madrid Institute for Advanced Studies in Nanoscience, IMDEA Nanociencia, Calle Faraday 9, Ciudad Universitaria de Cantoblanco, 28049 Madrid, Spain; ahmad.sousaraei@imdea.org (A.S.); santiago.casado@imdea.org (S.C.); jose.castro@imdea.org (J.R.C.-S.); juan.cabanillas@imdea.org (J.C.-G.); 3 REQUIMTE-LAQV & Department of Chemistry and Biochemistry, Faculty of Sciences, University of Porto, 4169-007 Porto, Portugal; josenmalmeida@gmail.com (J.A.); cpaqueiros@gmail.com (C.Q.); l.cunha.silva@fc.up.pt (L.C.-S.); ana.silva@fc.up.pt (A.M.G.S.)

**Keywords:** metal-organic frameworks, gas sensors, soft-imprinting, nitroaromatics, explosive detection

## Abstract

A novel technique for the creation of metal-organic framework (MOF) films based on soft-imprinting and their use as gas sensors was developed. The microporous MOF material [Zn_2_(bpdc)_2_(bpee)] (bpdc = 4,4′-biphenyldicarboxylate; bpee = 1,2-bipyridylethene) was synthesized solvothermally and activated by removing the occluded solvent molecules from its inner channels. MOF particles were characterized by powder X-ray diffraction and fluorescence spectroscopy, showing high crystallinity and intense photoluminescence. Scanning electron microscope images revealed that MOF crystals were mainly in the form of microneedles with a high surface-to-volume ratio, which together with the high porosity of the material enhances its interaction with gas molecules. MOF crystals were soft-imprinted into cellulose acetate (CA) films on quartz at different pressures. Atomic force microscope images of soft-imprinted films showed that MOF crystals were partially embedded into the CA. With this procedure, mechanically stable films were created, with crystals protruding from the CA surface and therefore available for incoming gas molecules. The sensing properties of the films were assessed by exposing them to saturated atmospheres of 2,4-dinitrotoluene, which resulted in a substantial quenching of the fluorescence after few seconds. The soft-imprinted MOF films on CA/quartz exhibit good sensing capabilities for the detection of nitroaromatics, which was attributed to the MOF sensitivity and to the novel and more efficient film processing method based on soft-imprinting.

## 1. Introduction

Metal-organic frameworks (MOFs) are crystalline organic-inorganic porous materials comprised of metal atoms or clusters coordinated by organic ligands to form extended coordination networks. The stability, high porosity, and easy à la carte synthesis of these materials are some of their key properties, warranting extensive use in many technological areas [1]. Gas storage and separation [2,3,4], catalysis [5,6], and chemical sensing [7,8,9] are just a few of the increasing number of applications benefitting from the versatility of these materials.

Owing to their optical properties and internal channels that provide them with an elevated porosity, microporous luminescent MOFs represent an ideal choice for the fabrication of gas-sensing devices based on fluorescence [7,10]. Explosives sensing is one of the most promising applications of this family of materials, which has attracted great attention for anti-terrorism operations, homeland security, and environmental protection in contaminated areas [11]. In this way, a number of works have studied the interaction between MOFs and nitroaromatics, such as 2,4-dinitrotoluene (DNT), precursor of 2,4,6-trinitrotoluene and part of the composition of explosives based in the latter [12,13,14].

In the search for solid-state sensors, researchers have deposited MOFs onto solid substrates in different ways over the last few years. MOFs cannot be easily dissolved and recrystallized without losing their particular structure, hence some authors report the suspension of the material for its later deposition as dip-coated [15] or spin-coated [16] films. These techniques have the main advantage of being fast and straightforward, but often do not offer accurate control over the preparation of the films and may inherently result in substantial waste of material. The Langmuir-Blodgett technique has also been reported as an option for the creation of MOF films [17,18], with the drawback of requiring either the presence of specific functional groups in the material for the formation of films at the air-water interface, or the addition of host molecules such as polymers. MOFs can also be grown on solid substrates following specific and adapted synthetic routes, which is a rather laborious process [19]. To our knowledge, one of the most common methods for the creation of MOF films involves, surprisingly, the use of adhesive tape [12,13,20]. Double-sided or electrical tape are among the preferred materials for the adhesion of MOF particles to solid substrates, usually glass or quartz. Although straightforward and affordable, this method present two main drawbacks: (i) first, there is a lack of control over the film and (ii) second, adhesive tape is not optically passive, but generates fluorescence upon UV photoexcitation, which is also responsible for solvent release which could potentially interact with the MOF.

**Figure 1:**
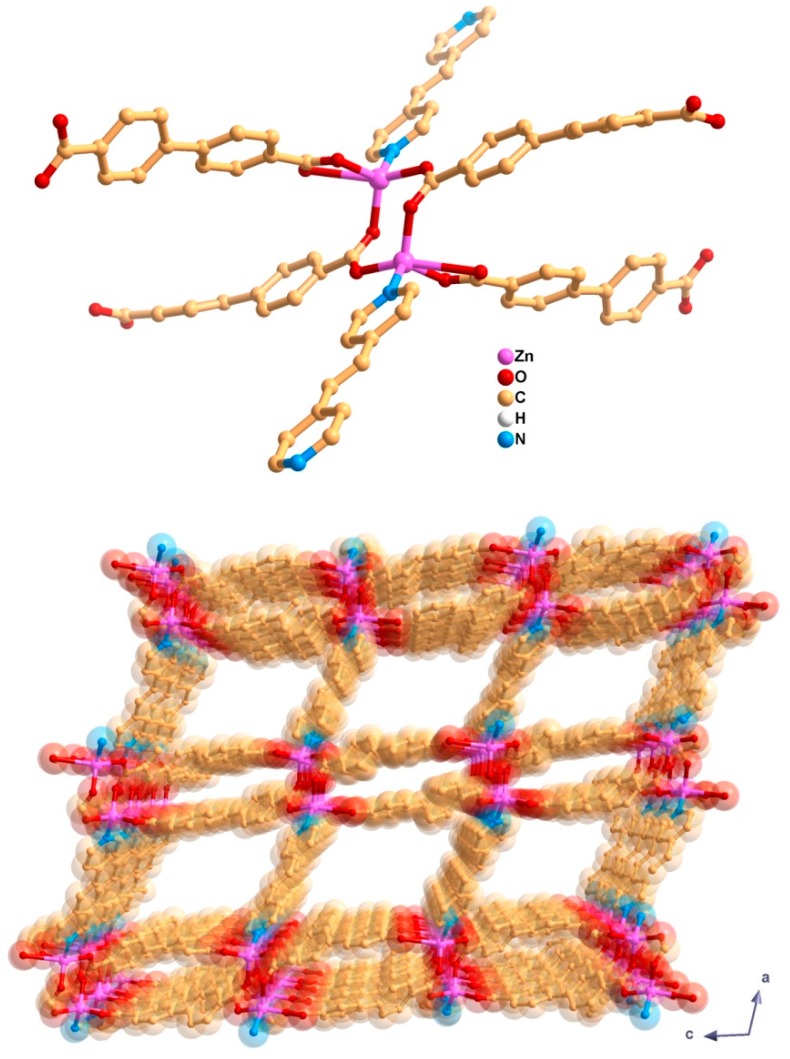
Selected structural features of [Zn_2_(bpdc)_2_(bpee)]: the secondary building unit showing the coordination environment of the Zn(II) centers (**top**) and crystalline packing arrangement revealing channels along the *b*-axis of the unit cell (**bottom**). For clarity, the H-atoms are omitted. The images were prepared from the original CIF file deposited in the Cambridge Structural Database (entry code COWPOU).

Here, we use the soft-imprinting technique for the deposition of a microporous MOF onto thin films. Cellulose acetate (CA) films with controlled thickness previously prepared by spin-coating on quartz are used as substrates. This technique allows the replication of nanometric features in thin films [21], into which MOF particles can be embedded with an improved film stability [22]. We aim to incorporate microcrystals of the MOF material [Zn_2_(bpdc)_2_(bpee)] (Figure 1; bpdc = 4,4′-biphenyldicarboxylate; bpee = 1,2-bipyridylethene) into CA films by embedding them only partially and leaving a portion on top of the surface in order to favor their interaction with gas molecules in the proximity of the film. With these films, we will assess the sensing capabilities of the microporous MOF towards DNT gas. This MOF has been reported to be sensitive to DNT and other nitroaromatics through fluorescence quenching [12,20], showing promising behavior for its implementation as an explosives sensor. We synthesize and activate [Zn_2_(bpdc)_2_(bpee)] for gas sensing by liberating its internal channels from solvent molecules following an improved method. Furthermore, we compare the soft-imprinting technique with the frequently used strategy based on the application of adhesive tape, aiming to improve the existing deposition methods, with a focus on the creation of MOF-based sensors for the detection of nitroaromatic explosives.

## 2. Results and Discussion

### 2.1. Synthesis and Characterization

**Figure 2:**
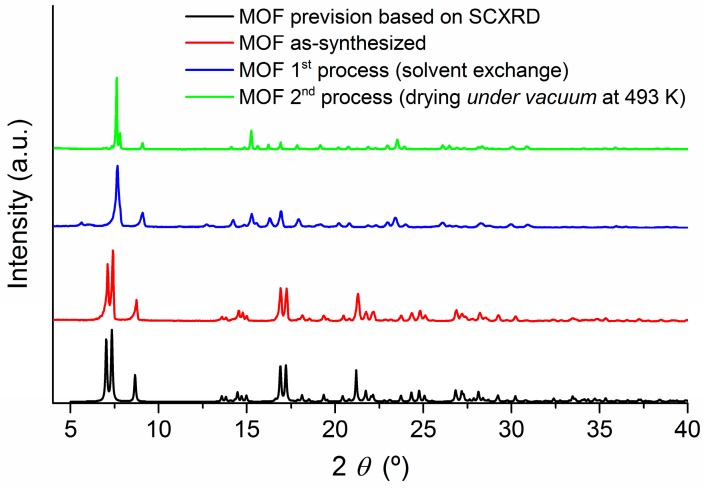
Powder X-ray diffraction patterns of the simulated pattern based on the single crystal structure of [Zn_2_(bpdc)_2_(bpee)] (**black**), the as-synthesized metal-organic framework (MOF) (**red**), a MOF sample after the first process—solvent exchange (**blue**)—and a MOF sample obtained after the second process—drying under vacuum at 493 K for 5 h (**green**).

The microporous MOF was prepared by a solvothermal reaction involving the coordination of two highly conjugated ligands, H_2_bpdc and bpee, with the Zn(II) metal ion. As can be seen in the powder X-ray diffraction (PXRD) analysis (Figure 2) the as-synthesized PXRD pattern (red line) showed high similarities with the simulation based on single crystal X-ray diffraction (SCXRD) database (black line), indicating the successful synthesis of the material. This is in agreement with the results published by Lan et al. [12].

As described by Lan et al. [12], the MOF structure contains roughly rectangular 1D channels in which dimethylformamide (DMF) solvent molecules are encapsulated. To activate this material, the DMF molecules need to be removed, releasing the channels (pores) and thus making it suitable for detection purposes. Our initial attempt to activate the material involved the treatment in CH_3_OH (three days) and CH_2_Cl_2_ (four days) for solvent exchange, followed by a drying period under vacuum at room temperature. Following this protocol, the PXRD analysis revealed that the intended MOF was obtained (Figure 2, blue line). However, our initial studies in gas detection showed a much lower sensitivity than reported, which we attributed to the material not being properly activated because the pores of the MOF had not been fully released from the DMF molecules, hindering the gas diffusion through the sensing material. Considering this, an alternative activation strategy to remove the uncoordinated DMF was tested, which involved the solvent exchange and a thermal drying procedure at 493 K under vacuum (30 mbar) for 5 h. In terms of PXRD analysis, Figure 2 presents the comparison for the MOF obtained from the first process of DMF removal (solvent exchange) and the second one (drying under vacuum at 493 K). The crystallinity and main structural features of the material were maintained after both processes, which correspond to the activation of the MOF. Apparently, the activation of the MOF by the removal of DMF molecules from the pores causes small structural changes, as reflected by the few alterations verified in the PXRD patterns. However, it is evident that the MOF material maintains considerable permanent porosity, allowing its potential activity in sensing.

**Figure 3:**
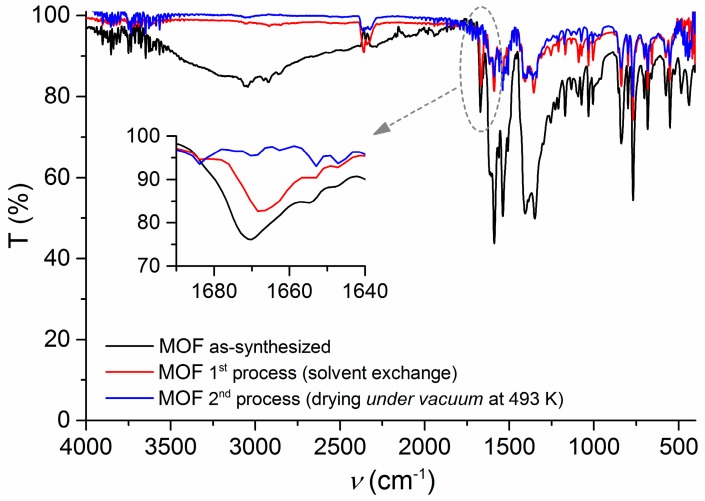
FTIR spectra of [Zn_2_(bpdc)_2_(bpee)] obtained from different conditions: as-synthesized (**black**), after the first process—solvent exchange (**red**)—and after the second—drying under vacuum at 493 K for 5 h (**blue**).

The incomplete elimination of DMF from the MOF after the solvent exchange was confirmed by FTIR spectroscopy. The presence of a characteristic C=O stretching peak at ~1670 cm^−1^ proved the existence of DMF molecules occluded into the material (Figure 3) [23]. Upon drying the MOF under vacuum at 493 K, DMF in the MOF was efficiently removed, as supported by the disappearance of the DMF C=O stretching peak from the FTIR spectrum of the desolvated sample (Figure 3). This procedure for DMF removal (pore activation) present a significant advantage in comparison with the reported one, as it took less than three days to obtain the MOF, instead of the seven days reported by Lan et al. [12]. The material prepared using the second process was the one used for the gas sensing tests due to its better performance in terms of sensitivity and gas diffusion.

**Figure 4:**
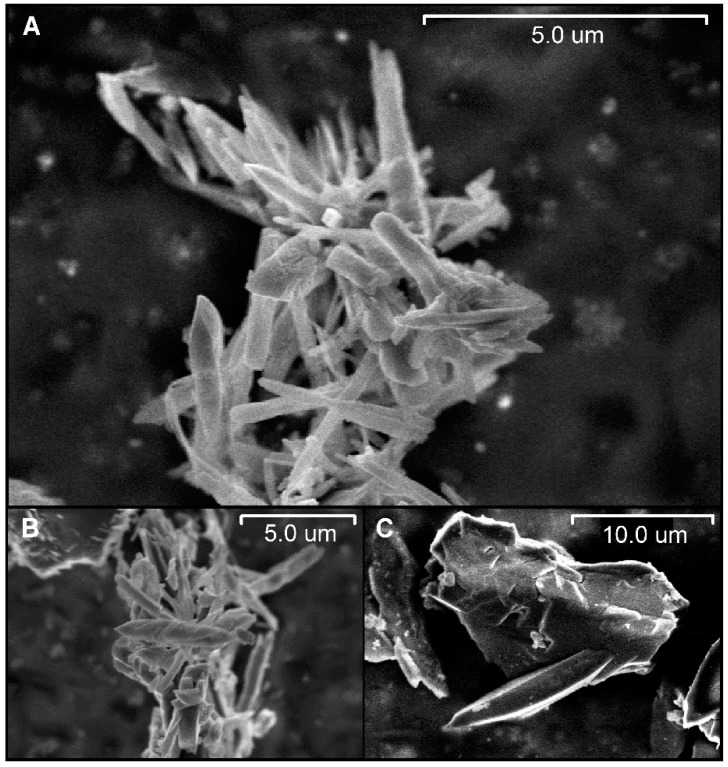
Scanning electron microscope images of [Zn_2_(bpdc)_2_(bpee)] powder. (**A**,**B**) [Zn_2_(bpdc)_2_(bpee)] microneedles; (**C**) [Zn_2_(bpdc)_2_(bpee)] aggregates.

SEM images of MOF showed the material in the form of microneedles alongside bigger aggregates with no particular shape (Figure 4). Such an arrangement leads to a high surface-to-volume ratio due to the small diameter of the microneedles, favoring the gas-sensing capabilities of our material due to the increase in exposed surface area. This adds to the fact that the MOF is highly porous owing to its internal channels [12,20], hence allowing easy diffusion of gas molecules through the internal structures and facilitating their accessibility to the material active sites.

### 2.2. Soft-Imprinted MOF Films

**Figure 5:**
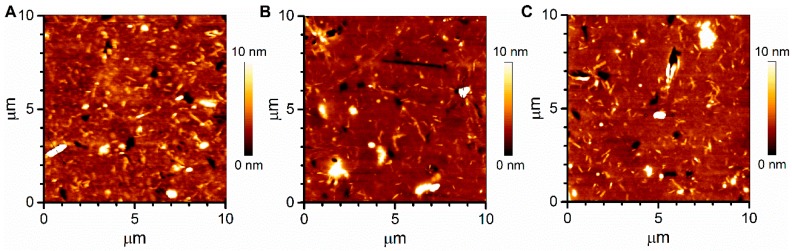
Atomic force microscope images of soft-imprinted [Zn_2_(bpdc)_2_(bpee)] powder on CA/quartz substrates prepared at (**A**) 2; (**B**) 4; and (**C**) 6 bar.

Soft-imprinting of MOF on CA-coated quartz substrates (CA thickness: 200 nm) with an applied pressure of 2, 4, and 6 bar resulted in MOF particles partially immersed into the CA film, as can be seen in atomic force microscope (AFM) images (Figure 5). In general, MOF crystals were dispersed on the surface and embedded but not covered by the CA, clearly protruding from the film. According to the images, individual microneedles were found, lying flat on the surface, along with bigger aggregates. In the analyzed regions, the average height of the microneedles from the CA surface was found to be around 5 nm, while that of the aggregates was approximately 95 nm. Further characterization of the films was performed by analyzing the roughness of the AFM images. Root mean square roughness for all samples was similar and in the order of 5 nm, indicating on one hand that the three different pressures applied to imprint the MOF led to films with similar surface characteristics. On the other hand, the roughness of the films points at the inhomogeneity of their surfaces, which can be beneficial for gas-sensing purposes due to a high surface area to volume ratio and improved access of gas molecules towards the active sites of the films, i.e., MOF particles. For comparison, AFM images of a pure CA film were also analyzed, revealing a smooth and even surface prior to the soft-imprinting of MOF (Figure S1). The CA layer was found to efficently retain the MOF particles, providing mechanical stability to the sensor. By using a thin CA film, we aimed to only partially embed the MOF particles in order to leave part of them available to the gases in the environment, hence facilitating gas diffusion through them into the portion submerged into the CA, whilst assuring MOF immobilization on the substrate. In this way, a compromise between adhesion of the MOF to the substrate and accessibility of gas molecules was achieved. Total immersion of MOF particles into CA would probably further increase film stability, but with the undesired outcome of hindered access of gas molecules towards the interior of the sensing material.

**Figure 6:**
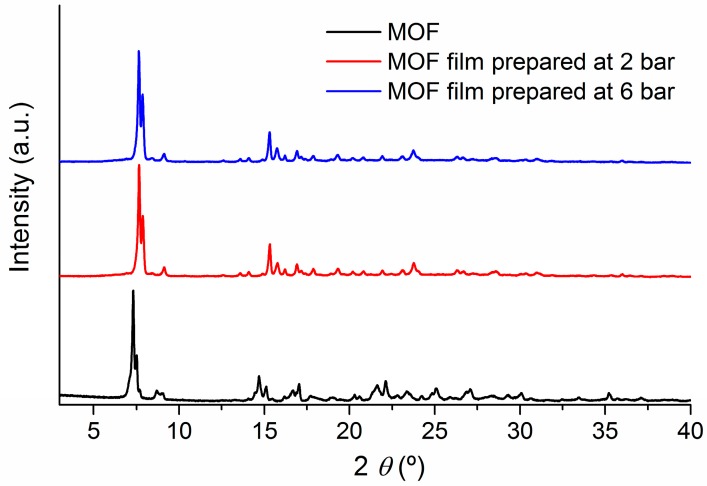
Powder X-ray diffraction patterns of [Zn_2_(bpdc)_2_(bpee)] (**black**) and films prepared by soft-imprinting on cellulose acetate at 2 (**red**) and 6 bar (**blue**).

To analyze whether the pressures applied during the soft-imprinting process affected the crystallinity of the MOF, we compared the PXRD patterns of MOF activated (desolvated) at 493 K under vacuum for 5 h with its corresponding films at 2, 4, and 6 bar. In Figure 6, extreme cases of low (2 bar) and high (6 bar) pressure are shown. As can be seen, in both cases the PXRD patterns were very similar, despite the peaks shift verified in the diffractograms of the two films relative to the MOF powder, which corresponds to an experimental misalignment due to the distinct morphology of the analyzed samples (films and powder). These observations indicating that the crystallinity was retained after the soft-imprinting process, and no observable differences occur in the preparations between 2 and 6 bar. With this outcome, the suitability of soft-imprinting as a technique for the processing of MOFs onto solid substrates is confirmed.

### 2.3. Explosive Vapor Sensing

MOF powder was first deposited on glass substrates using either double-sided tape or the adhesive residue left after peeling off the tape. Similar procedures for the creation of MOF films can be found in the literature [12]. We discarded double-sided tape as method to adhere the MOF to the substrate due to the strong photoluminescence in the region of 400–500 nm, coinciding with the emission band of the MOF (Figure S2). Besides, solvents and other compounds contained in the adhesive tape may interact with the sensing material, altering its response towards the analytes to be detected. These drawbacks were minimized by using only the adhesive residue left by the tape, which showed no photoluminescence (Figure S2) and allowed the fabrication of uniform MOF thin films. These films were exposed to DNT, resulting in an important quenching of their fluorescence (Figure S3). This proves the potential of the developed samples as an explosives sensor, in agreement with the results published by other authors [12]. However, the adhesive-residue method for the deposition of the MOF showed very little stability, as MOF particles were only partially adhered to the substrate. Even with extreme care, manipulation of the films led to MOF particles dropping onto every surface. Successive introduction and removal of the films from the fluorimeter caused a progressive loss in fluorescence intensity due to a decrease in the amount of surface covered by MOF particles that was even visible to the naked eye (Figure S4). This instability limits the possibilities of the films to be used as sensors in commercial applications.

**Figure 7:**
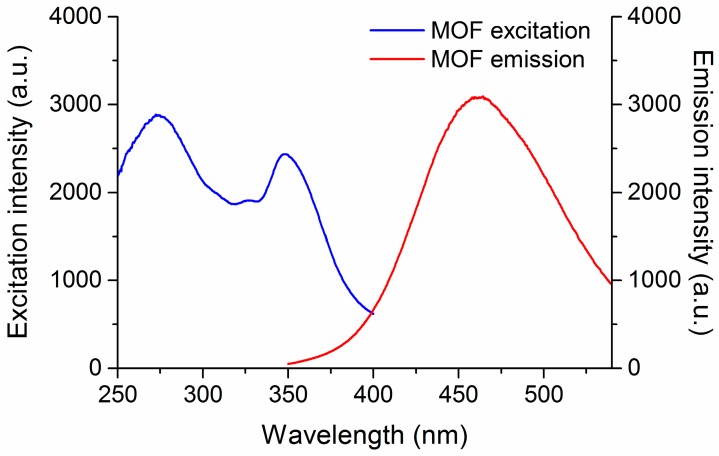
Photoluminescence spectra (λ_exc_ = 280 nm) and excitation spectra (λ_em_ = 460 nm) of [Zn_2_(bpdc)_2_(bpee)] powder deposited by soft-imprinting.

As an alternative, soft-imprinted MOF films on CA-coated quartz were used for the detection of DNT. Films prepared at 4 bar were chosen for this purpose. Photoluminescence spectra of activated MOF powder deposited by soft-imprinting showed an intense emission band at 460 nm when excited at 280 nm (Figure 7, red line). This excitation wavelength was chosen based on the excitation spectra (Figure 7, blue line), which showed an excitation region from 250 nm to 370 nm. The excitation at 280 nm provided the highest emission among the different wavelengths tested, hence maximizing the intensity of the band to be monitored in subsequent sensing experiments. Identical results were obtained by depositing MOF powder on glass with the adhesive residue left by a double-sided-tape. The high emission intensity observed in the photoluminescence spectrum of the sample suggests its suitability as a fluorescent probe.

**Figure 8:**
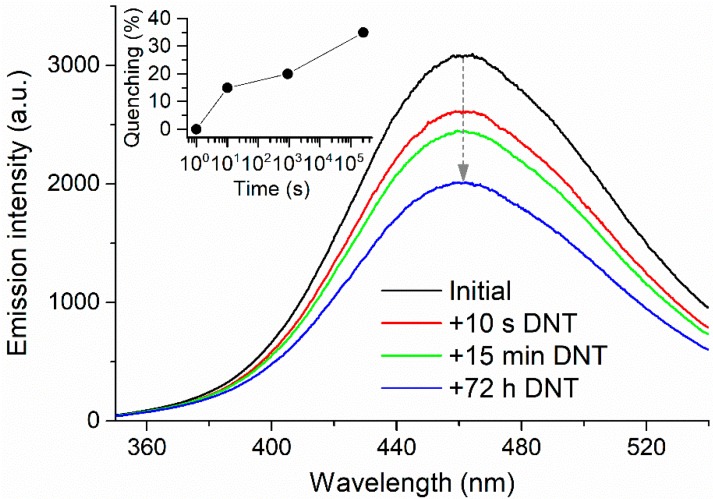
Exposure of [Zn_2_(bpdc)_2_(bpee)] soft-imprinted on CA/quartz at 4 bar to DNT vapors. Inset: quenching percentages for each exposure time.

The exposure of the films to DNT vapors resulted in a substantial quenching of the typical fluorescence of [Zn_2_(bpdc)_2_(bpee)] (Figure 8). This quenching has been attributed to the presence of two -NO_2_ electron-withdrawing functional groups in the structure of DNT. As a result, the nitroaromatic π system would be electron-depleted and the molecule would behave as a π acceptor upon interaction with the organic ligands of the MOF [20]. We quantified the quenching of fluorescence (%) as $\left(I_{0}-I\right)/I_{0}\times 100$, in which $I_{0}$ is the maximum fluorescence intensity of the sample and I is the corresponding maximum intensity after exposure to DNT (Figure 8, inset). The emission intensity was monitored at the wavelength of maximum emission (460 nm). Mean fluorescence quenching for a 10-s exposure was found to be 15%, indicating the fast response of our sensor towards DNT. On average, all films responded equally to the nitroaromatic irrespective of the pressure applied during the soft-imprinting procedure (Figure S5), which suggests an elevated reproducibility of the method. However, it must be noted that this quenching was possibly underestimated due to experimental constraints. DNT exhibits extremely low vapor pressures, requiring long times for the generation of saturated environments. In our case, saturation was ensured by generating DNT headspace in a small vial during a long period of time (four days). However, the dilution of the saturated headspace during the manipulation of the vial for the introduction of the film cannot be discarded. This would explain different quenching values found by other authors [12], besides other experimental variations or the existence of undisclosed preparation details in other publications that might affect the sensing capabilities of the MOF or its interaction with DNT.

Our DNT sensor benefited from the good sensing capabilities shown by the MOF and from the enhanced stability of the films prepared by soft-imprinting. This outcome confirms the suitability of [Zn_2_(bpdc)_2_(bpee)] for the detection of nitroaromatics. Besides, the soft-imprinting technique emerges as an ideal tool for the controlled and reproducible processing of MOFs or similar materials onto solid substrates. In our view, these promising results pave the way for an improved method for the fabrication of MOF sensors. To do this, the soft-imprinting technique still needs to be refined by analyzing all variables in the process and by extending the scope of this study to other MOFs and their exposures to other analytes. Besides, efforts are being made to establish a recovery procedure for the reutilization of the sensors. Meanwhile, the potential applications of our films are oriented towards disposable sensors. Last but not least, the system for the exposure to nitroaromatics needs to be perfected. Due to their low volatility, the typical procedure found in the literature merely consists of the insertion of the sensor into a sealed vial containing a saturated headspace generated by the desired compound [12,14,20]. With this technique, which we also followed in our experiments, the concentration of nitroaromatics cannot be controlled. Our current experimental efforts are working towards the implementation of a system for the generation of controlled atmospheres of nitroaromatics, intended to be able to quantify the responses of our sensors.

## 3. Materials and Methods

### 3.1. Chemicals and General Methods

All solvents and starting materials for synthesis were commercially available and were used as received. Fourier transform infrared spectra (FTIR) were collected using a Shimadzu IRAffinity-1 from 4000–400 cm^−1^. PXRD patterns were collected at room temperature using an Empyrean PANalytical diffractometer (CuKα_1,2_ radiation, λ_1_ = 1.540598 Å and λ_2_ = 1.544426 Å) equipped with a PIXcel 1D detector and a flat-plate sample holder in a Bragg–Brentano para-focusing optics configuration (45 kV, 40 mA). Intensity data were obtained by the step counting method (step: 0.02°) in continuous mode in the approximate range 3.0° ≤ 2θ ≤ 50°. Scanning electron microscope (SEM) images were acquired using a Philips CM-200 (200 kV). Fluorescence spectra for the characterization of the MOF and for DNT sensing were collected using a Hitachi F-7000 Fluorescence Spectrophotometer (Hitachi High Technologies, Krefeld, Germany). Fluorescence spectra of films containing [Zn_2_(bpdc)_2_(bpee)] were obtained by using a sample holder for solid samples. AFM images were acquired by means of a JPK NanoWizard II AFM functioning in contact mode, connected to a Nikon Eclipse Ti inverted optical microscope. MikroMasch HQ:XSC11/Al BS cantilevers of 0.2 N/m spring constant and 15 kHz resonant frequency were used in air conditions.

### 3.2. Synthesis of [Zn_2_(bpdc)_2_(bpee)] MOFs

[Zn_2_(bpdc)_2_(bpee)] was synthesized using solvothermal conditions as reported by Lan et al. [12], with a few slight modifications. Briefly, a mixture of Zn(NO_3_)_2_·6H_2_O (0.0921 g, 0.31 mmol), 4,4′-biphenyldicarboxylic acid (H_2_bpdc, 0.0734 g, 0.30 mmol), and 1,2-bipyridylethene (bpee, 0.0554 g, 0.30 mmol) in DMF (15 mL) was placed in a 23-mL teflon-lined reactor, which was sealed in a stainless-steel vessel. A total of 20 reactors were prepared and placed in the oven, at 438 K for three days, to afford the MOF material as block-shaped crystals. After cooling, the resultant solid was filtered and stirred in DMF (20 mL) at 338 K for 24 h to remove to unreacted ligands. Afterwards, the precipitant was separated and immersed in CH_3_OH (20 mL), which was stirred at 343 K for 45 min. The product was collected by centrifugation (10 min, at 3500 rpm) and suspended in CH_2_Cl_2_ (20 mL). After filtration, the material was activated at 493 K under vacuum (30 mbar) for 5 h, to give 1.12 g (24% yield) of [Zn_2_(bpdc)_2_(bpee)].

### 3.3. Fabrication of Adhesive Tape-Based MOF Films

Glass slides were cut to size (26 × 8 mm^2^) and carefully cleaned with acetone and lint-free wipes. Double-sided tape was then applied to the lower part of the glass substrates and manually pressed until complete adhesion was ensured. For the preparation of thin layers, the tape was removed after a few minutes, leaving a small amount of adhesive residue onto the glass substrate. In the case of thick layers, the double-sided tape was not removed at any time during the preparation procedure. Subsequently, for the fabrication of both thin and thick films, the substrates containing either the adhesive residue or the double-sided tape were covered with [Zn_2_(bpdc)_2_(bpee)] MOF powder, resulting in the substrates coated with a fine layer of crystals. Afterwards, the films were gently tapped while facing down in order to remove any crystals that were not adhered well to the surface.

### 3.4. Fabrication of Soft-Imprinted MOF/CA Films

First, a solution of CA (40 mg/mL, MW = 61,000, resulting refractive index of the film *n* = 1.48 at 570 nm, Sigma Aldrich, Madrid, Spain) in 4-hydroxy-4-methyl-2-penthanon (diacetone alcohol, Sigma Aldrich), was spin-coated at 3000 rpm for 1 min, with the acceleration set at 12,000 rpm/s, onto a quartz substrate. The thickness of the samples was measured using a mechanical profilometer Bruker Dektak XT with a 2.5-mm radio stylus. The applied strength was the minimum possible (around 1 mg), and the length of analysis was 7500 μm. Scratches were performed on the surface of the sample prior to the scan. All measurements were repeated five times and the results averaged. The CA films used in this work had an average thickness of 200 nm, which was determined by the concentration of CA solution (Figure S6). [Zn_2_(bpdc)_2_(bpee)] in the form of powder was deposited manually on top of the CA film and an identical CA/quartz system was used to encapsulate the MOFs during the soft-imprint process. The films were imprinted by means of a Compact Nanoimprint Tool (CNI Tool, NILT, Kongens Lyngby, Denmark). During the hot embossing process, the substrates were heated to 413 K with applied pressures of 2, 4, and 6 bar for 1200 s each. The samples were subsequently allowed to cool down to a temperature of 343 K before the pressure was released and the imprinted substrate removed. As a result, two CA/quartz substrates with a similar quantity of partially embedded MOFs were obtained. Given the reduced thickness of the CA film and micron-size of [Zn_2_(bpdc)_2_(bpee)], most of the material was exposed, allowing the sensing experiments.

### 3.5. Exposure to Explosives

Prior to the sensing experiments, a small open vial containing DNT (Sigma Aldrich, Madrid, Spain) was introduced in another vial hermetically sealed and allowed to evaporate for four days at laboratory temperature until headspace saturation. Such long times were chosen due to the low vapor pressure of DNT. Similar procedures for the creation of saturated explosive atmospheres can be found in the literature [12]. The exposure to explosives was achieved by introducing the sensor film into the bigger vial for the required time. Subsequently, the film was rapidly placed in the sample holder of the fluorimeter and the corresponding measurement was performed immediately. The fluorescence of the films was measured in different positions to ensure reproducibility of the method, and the quenching of fluorescence upon exposure to DNT was calculated with respect to the corresponding initial spectrum for each position.

## 4. Conclusions

A microporous [Zn_2_(bpdc)_2_(bpee)] MOF was successfully prepared by a solvothermal reaction involving the coordination of the ligands H_2_bpdc and bpee with the Zn(II) metal ion. The powder XRD analysis revealed that a highly porous structure with internal 1D channels was obtained. FTIR showed that DMF molecules were occluded into the MOF channels. These uncoordinated solvent molecules were removed by solvent exchange and a thermal drying procedure under vacuum that proved to be successful according to FTIR spectra, besides being faster than other reported techniques. SEM images showed that MOF particles were arranged mostly as crystalline microneedles, resulting in a high surface area to volume ratio that enhanced the gas molecules’ interaction with the material. MOF particles were successfully soft-imprinted onto CA films at different pressures, resulting in all cases in partially embedded particles, according to AFM images. This arrangement favored both film stability, due to CA holding MOF particles in place, and gas accessibility to the MOF active sites, owing to the particles partially protruding from the CA film. Fluorescence spectra of soft-imprinted MOF films showed a high emission intensity, indicating the suitability of the MOF to be used as a fluorescent probe. Exposure of the soft-imprinted films to DNT resulted in a substantial quenching of the fluorescence after a few seconds. Overall, it can be concluded that [Zn_2_(bpdc)_2_(bpee)] soft-imprinted films on CA exhibit good sensing capabilities as candidates for the detection of nitroaromatics, owing to the MOF sensitivity and to the novel and more efficient film processing method based on soft-imprinting.

## Supplementary Materials

The following are available online at www.mdpi.com/1996-1944/10/9/992/s1, Figure S1: AFM image of a pure CA film on quartz prepared by spin-coating; Figure S2: Photoluminescence spectra of double-sided tape, adhesive residue after peeling off the tape, MOF adhered to the tape and MOF adhered to the adhesive residue; Figure S3: Photoluminescence spectra of an MOF film prepared with the adhesive residue left by an adhesive tape and after its exposure to a DNT saturated environment; Figure S4: Photoluminescence spectra and pictures of an MOF film prepared with the adhesive residue left by an adhesive tape before and after typical experimental manipulation; Figure S5: Quenching percentages for films prepared at 2, 4, and 6 bar; Figure S6: Thickness of CA films prepared from CA solutions at different concentrations.
