# Supplementary material for: Preparation of Luminescent Metal-Organic Framework Films by Soft-Imprinting for 2,4-Dinitrotoluene Sensing
Source: Materials (Basel). 2017 Aug 25;10(9):992. doi: 10.3390/ma10090992 (PMC5615647; doi:10.3390/ma10090992)
Supplement: Supplementary file 1 [file materials-10-00992-s001.pdf]

Preparation of Luminescent Metal-Organic Framework Films by Soft-Imprinting for 2,4-Dinitrotoluene Sensing - Supplementary Materials

Javier Roales,[1] Francisco G. Moscoso,[1] Francisco Gámez,[1] Tânia Lopes-Costa,[1] Ahmad Sousaraei,[2] Santiago Casado,[2] Jose R. Castro-Smirnov,[2] Juan Cabanillas-Gonzalez,[2] José Almeida,[3] Carla Queirós,[3] Luís Cunha-Silva,[3] Ana M. G. Silva [3] and José M. Pedrosa[1]

[1] *Departamento de Sistemas Físicos, Químicos y Naturales. Universidad Pablo de Olavide, Ctra. Utrera Km. 1, 41013 Sevilla, Spain*

[2] *Madrid Institute for Advanced Studies in Nanoscience, IMDEA Nanociencia, Calle Faraday 9, Ciudad Universitaria de Cantoblanco, 28049 Madrid, Spain*

[3] *REQUIMTE-LAQV & Department of Chemistry and Biochemistry, Faculty of Sciences, University of Porto, 4169-007 Porto, Portugal*

1. AFM analysis of a pure CA film

The AFM analysis of a pure CA film spin-coated on quartz without MOF crystals embedded showed an even and smooth surface without protruding domains (Figure S1), indicating that the irregularities found in the soft-imprinted films were effectively MOF particles.

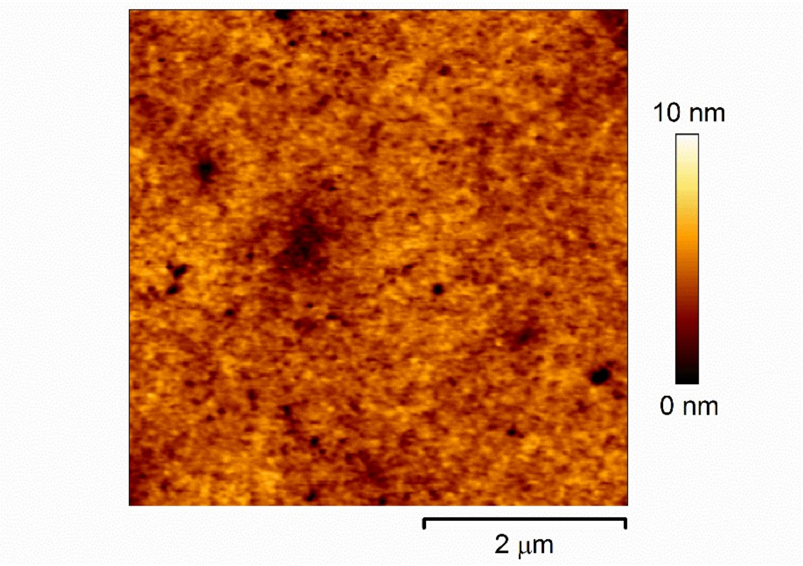

Figure S1. AFM image of a pure CA film on quartz prepared by spin-coating.

2. MOF film preparation using adhesive tape

[$Zn_2(bpdc)_2(bpee)$] MOF films prepared with double-sided tape applied onto glass substrates showed intense photoluminescence when excited at 280 nm (Figure S2, green line). We discovered that there was a contribution of the tape itself to the fluorescence of the films (Figure S2, black line), hence rendering the films unusable for our purposes due to the impossibility of differentiating between the fluorescence of the support and that of the MOF. This procedure was then discarded and we prepared the MOF films with the adhesive residue left after peeling off the tape from the glass substrate, which showed no emission at all (Figure S2, red line), leading to a less intense but still adequate emission intensity (Figure S2, blue line).

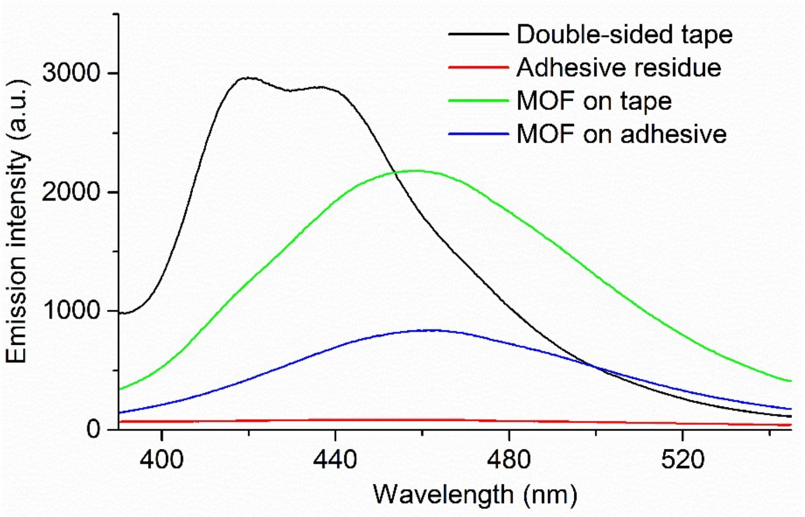

Figure S2. Photoluminescence spectra of double-sided tape, adhesive residue after peeling off the tape, [$Zn_2(bpdc)_2(bpee)$] adhered to the tape and [$Zn_2(bpdc)_2(bpee)$] adhered to the adhesive residue. All samples were excited at 280 nm.

3. Exposure of MOF films based on adhesive tape residue to DNT

The exposure of a MOF film based on the adhesive residue left by an adhesive tape to DNT resulted in the quenching of its fluorescence (Figure S3). This exposure was performed by inserting the film into a vial containing a saturated atmosphere in DNT.

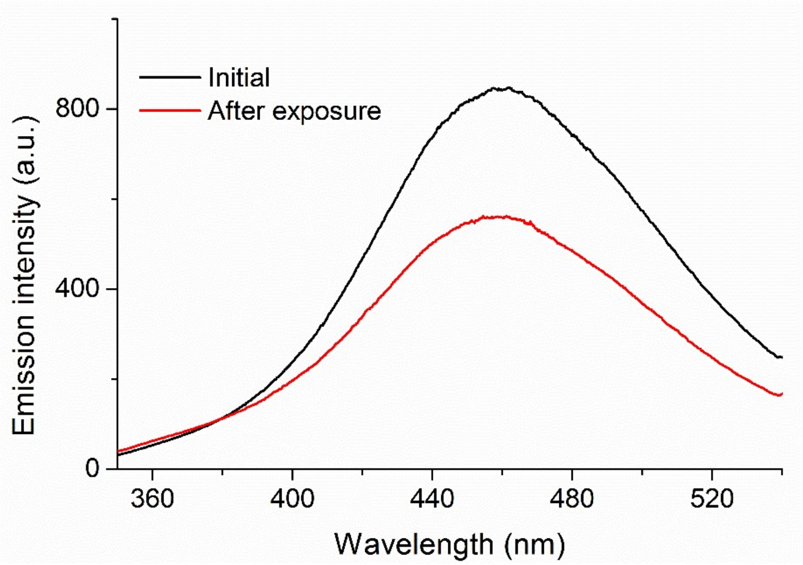

Figure S3. Photoluminescence spectra of a $[Zn_2(bpdc)_2(bpee)]$ film prepared with the adhesive residue left by an adhesive tape and after its exposure to a DNT saturated environment.

4. Stability of MOF films based on adhesive tape residue

Stability of tape-based films was assessed by recording their photoluminescence spectra immediately after preparation and after typical manipulation in the experiments, which involved insertion in the sample holder of the fluorimeter, posterior removal and storage in sealed vials between measurements. This manipulation led to the decrease of emission due to the loss of MOF particles in the surface (Figure S4). Such decrease in the amount of particles on the films was noticeable to the naked eye (see pictures in Figure S4).

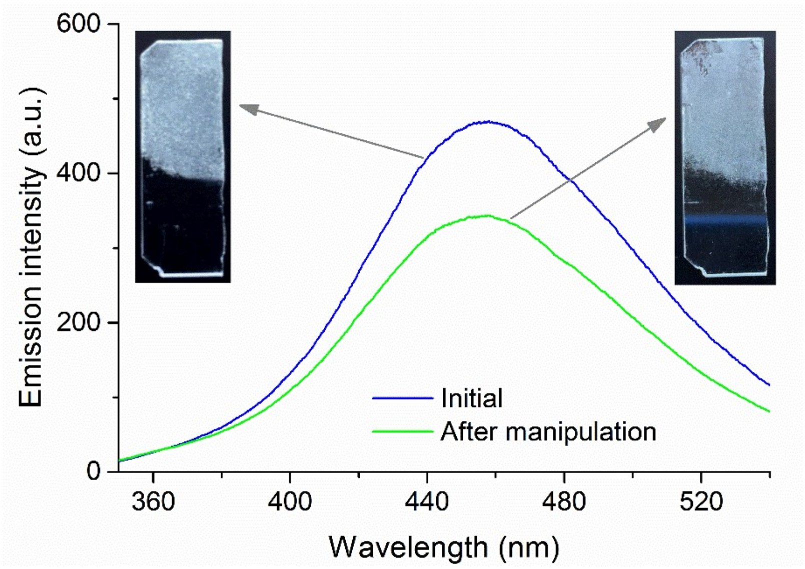

Figure S4. Photoluminescence spectra and pictures of a MOF film prepared with the adhesive residue left by an adhesive tape before (blue line and image on the left) and after typical experimental manipulation (green line and image on the right).

5. Quenching percentages for films prepared at 2, 4 and 6 bar

MOF films prepared by soft-imprinting were exposed to DNT and their sensing response was assessed by measuring the quenching of fluorescence in different positions for each film. On average, the comparison between films prepared at 2, 4 and 6 bar showed no differences regarding the applied pressure (Figure S5), which indicates a high reproducibility of the method.

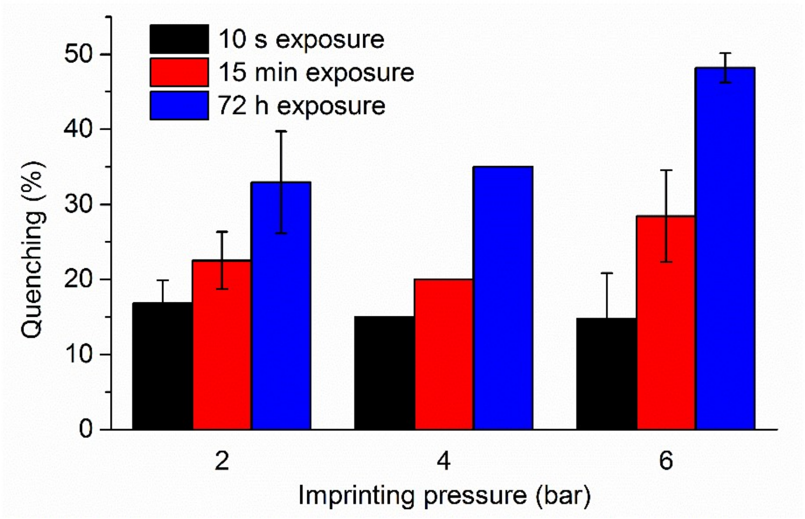

Figure S5. Quenching of fluorescence for films prepared at 2, 4 and 6 bar after their exposure to DNT.

6. CA film thickness

CA film thickness prior to imprinting of the MOF was assessed using a Bruker Dektak XT profilometer with a 2.5 mm radio stylus. Different concentrations of CA were used to study their effect on film thickness. All measurements were repeated 5 times and the results averaged. Film thickness increased clearly with CA concentration (Figure S6). For all subsequent experiments, we used films prepared at 40 mg/mL with a resulting thickness of 200 nm.

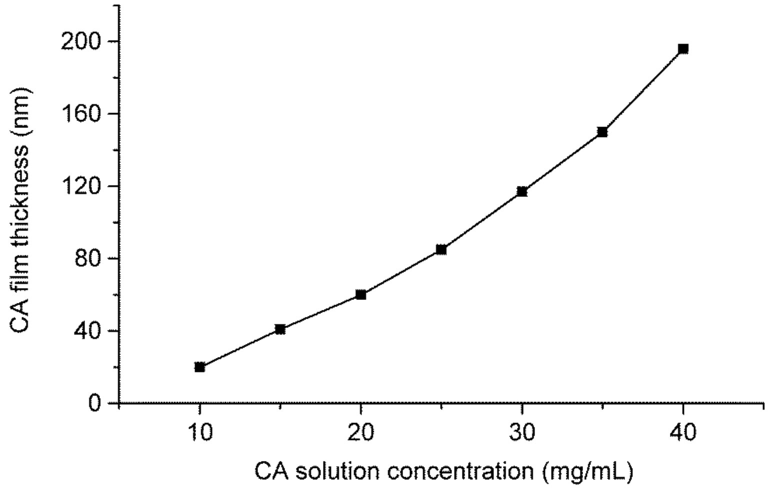

Figure S6. Thickness of CA films prepared from CA solutions at different concentrations.
